# Genomic signatures of host adaptation in group B *Salmonella enterica* ST416/ST417 from harbour porpoises

**DOI:** 10.1186/s13567-021-01001-0

**Published:** 2021-10-21

**Authors:** Arnar K. S. Sandholt, Aleksija Neimanis, Anna Roos, Jenny Eriksson, Robert Söderlund

**Affiliations:** 1 Department of Microbiology, National Veterinary Institute, Uppsala, Sweden; 2 Department of Pathology and Wildlife Diseases, National Veterinary Institute, Uppsala, Sweden; 3 Department of Environmental Research and Monitoring, Swedish Museum of Natural History, Stockholm, Sweden

**Keywords:** Pangenome analysis, cgMLST, salmonellosis, marine mammals, cetaceans, host bias, molecular epidemiology, extraintestinal infection, porpoise, *Phocoena*

## Abstract

A type of monophasic group B *Salmonella enterica* with the antigenic formula 4,12:a:- (“Fulica-like”) has been described as associated with harbour porpoises (*Phocoena phocoena*), most frequently recovered from lung samples. In the present study, lung tissue samples from 47 porpoises found along the Swedish coast or as bycatch in fishing nets were analysed, two of which were positive for *S. enterica*. Pneumonia due to the infection was considered the likely cause of death for one of the two animals. The recovered isolates were whole genome sequenced and found to belong to sequence type (ST) 416 and to be closely related to ST416/ST417 porpoise isolates from UK waters as determined by core-genome MLST. Serovars Bispebjerg, Fulica and Abortusequi were identified as distantly related to the porpoise isolates, but no close relatives from other host species were found. All ST416/417 isolates had extensive loss of function mutations in key *Salmonella* pathogenicity islands, but carried accessory genetic elements associated with extraintestinal infection such as iron uptake systems. Gene ontology and pathway analysis revealed reduced secondary metabolic capabilities and loss of function in terms of signalling and response to environmental cues, consistent with adaptation for the extraintestinal niche. A classification system based on machine learning identified ST416/417 as more invasive than classical gastrointestinal serovars. Genome analysis results are thus consistent with ST416/417 as a host-adapted and extraintestinal clonal population of *S. enterica*, which while found in porpoises without associated pathology can also cause severe opportunistic infections.

**Supplementary Information:**

The online version contains supplementary material available at 10.1186/s13567-021-01001-0.

## Introduction

The harbour porpoise (*Phocoena phocoena*) is a species of small cetaceans that inhabit coastal waters of the northern hemisphere. While it is abundant in the North Atlantic and the North Pacific, the Baltic Sea and Black Sea populations are critically endangered [1]. Because of their near-shore habitat, harbour porpoises are vulnerable to anthropogenic environmental disturbance including chemical and noise pollution and prey depletion as well as entanglement in fishing nets, especially in semi-enclosed seas such as the Baltic [1]. Such environmental stressors can weaken the immune system and leave animals more vulnerable to infection [2].

Harbour porpoises in Swedish waters can be classified into three populations: The North Sea, Belt Sea and Baltic Proper populations. Animals that are found dead are collected and examined to identify causes of death and other threats [3]. In a study of 98 stranded porpoises collected from 2008 to 2019, infectious disease was the second most common cause of death (17%) after bycatch and probable bycatch (25%). Infectious diseases included several cases of bacterial pneumonia in the presence of nematodes [3]. This is consistent with reports of a high frequency of pneumonia associated with parasites, bacterial infection, or both, among porpoises from the North Atlantic examined post-mortem [4–8].

A particular type of monophasic group B *Salmonella enterica* subsp. *enterica*, most often of sequence type (ST) 416 and with the antigenic formula 4,12:a:- (“Fulica-like”) has been described as exclusively associated with harbour porpoises in Scotland and England [9, 10]. This type of *Salmonella* occurs more frequently in lung samples than other tissues or faeces [11], and has also been recovered from porpoise lung worms [12] suggesting a possible mode of transmission. As low-grade *Salmonella* infections in the lungs appear to be prevalent among porpoises, it has been hypothesized that the infection becomes severe or life-threatening primarily in weakened individuals [11]. While most *S. enterica* are host generalists and cause self-limiting gastroenteritis in infected animals or humans, host adapted *Salmonella* serovars tend to infect one or a few host species, have a higher likelihood of causing extraintestinal infection, and more frequently establish a chronic carrier state in otherwise healthy infected animals, typical examples being *S*. Dublin in cattle, *S*. Choleraesuis in pigs and *S*. Gallinarum in poultry [13]. The evolution of a host adapted lineage of *Salmonella* from generalist ancestors appears to involve extensive degradation of genes not needed in the specialist niche with a corresponding loss of metabolic capability, as well as acquisition of mobile genetic elements with genes providing new traits [14–18]. Host and niche adaptation of the proposed porpoise-associated strains of *Salmonella* should therefore be evident in their genome sequences. In the present study we report the occurrence of *S. enterica* ST416 in lung samples from Swedish harbour porpoises and show extensive genomic evolution in these strains consistent with host adaptation and an increased capacity for causing extraintestinal infection.

## Materials and methods

### Animals, samples, and bacterial isolates

In Sweden, porpoises and several other mammalian and avian species of conservation interest are state property (“statens vilt” or “wildlife of the state”) and must be reported to the authorities if they are found dead. From 2008 to 2020, 142 porpoises found along the Swedish coast or incidentally killed as bycatch in fishing nets have been collected for necropsy examination within a collaborative program delivered by the Swedish National Veterinary Institute (SVA) and the Swedish Museum of Natural History. Almost all animals were stored frozen before investigation. Porpoises were examined following standardized protocols [19] to determine cause of death, investigate any abnormalities and collect tissues and data for other studies and archives [3]. Representative pieces of lung were fixed in 10% neutrally buffered formalin and processed and embedded in paraffin for microscopic examination. Sections (3–4 µm) were stained using Mayer’s hematoxylin and eosin [20]. Prior to 2018, any lesions consistent with possible bacterial infection were submitted for aerobic bacterial culture (*n* = 9 animals, of which lung was cultured in 5 animals) as described below. Since 2018, lung tissue from all porpoises that were not more than moderately decomposed were submitted for aerobic bacterial culture only (*n* = 21), for selective culture of *Salmonella* sp. (*n* = 15) or for both (*n* = 8). In total, lung tissue was cultured from 47 porpoises in this study. All bacteriology was performed by the Department of Microbiology, SVA. For aerobic bacterial culture samples were inoculated onto blood agar plates containing 5% horse blood and bromocresol purple lactose agar plates and held at 37 °C under aerobic conditions. In parallel, horse blood agar plates were grown at 37 °C in a 5% ± 1% CO_2_ incubator. Plates were inspected for growth at 24 and 48 h after inoculation. For selective culture, ISO 6579-1:2017 was followed with samples enriched in buffered peptone water and grown on modified semi-solid Rappaport–Vassiliadis (MSRV) agar, horse blood agar, brilliant green (BG) agar and xylose lysine deoxycholate (XLD) agar. Suspected *Salmonella* colonies were confirmed to species level by either real-time PCR [21] (in 2017) or by MALDI-TOF MS on a Bruker Biotyper instrument (in 2020) according to manufacturer instructions. Serotyping was performed with biochemical testing and slide agglutination, also according to ISO 6579-1:2017.

### Sequencing and reference data

Libraries were generated from 17-VLT002652 with a Nextera XT kit and sequencing performed on an Illumina MiSeq instrument as 2 × 250 bp paired-end. Additional long read data was generated using an Oxford Nanopore MinION sequencing device run with a R9.4.1 flow cell and a library created using the Rapid PCR barcoding kit. Sequencing of the second isolate 20-VLT001389 was performed by Illumina technology in the same way but as 250 + 60 bp paired-end due to technical issues with the reverse read. All three sequence datasets were a minimum of 100×. Short read data from 59 close relatives of the Swedish porpoise strains were identified by core genome multi-locus sequence typing (cgMLST) via Enterobase [22], together forming group 4372 at the hierarchical clustering level HC2000 as defined by the HierCC algorithm. Short read data from these isolates were downloaded from the European Nucleotide Archive for inclusion in downstream analysis. Short read data was quality checked using FastQC v0.11.8 and trimmed using Trimmomatic v0.39, with a sliding window of 4 bp and quality threshold of 20. Long read data was quality checked using FastQC and NanoPlot v1.32.1 [23] and filtered using Filtlong v0.2.0 keeping the best 900 Mbp of reads. The *S. enterica* type strain LT2 (*S*. Typhimurium) genome, GenBank NC_003197, was used as reference for read mapping, annotation, and analysis of *Salmonella* pathogenicity islands.

### Data analysis and visualization

Bowtie2 v2.3.5.1 was used to map short read data to the LT2 reference genome, and Samtools v1.9 was used for single nucleotide polymorphism (SNP) calling. The resulting VCF files were filtered using in-house R scripts (Additional file 4) and quality checked using the VCFR package v1.8.0. SNPEff v4.3 [24] was used to evaluate the impact of each SNP found. Gene Ontology (GO) categories impacted by the mutations were found using the Panther GO Enrichment tool [25] and the KEGG Mapper tool [26]. A machine learning algorithm developed by Wheeler et al. [27] was applied to read data from all 61 strains to assess the invasiveness of each and how it compared to known enteric and invasive *Salmonella* serovars. Unicycler v0.4.8 [28] with default settings was used to create a hybrid assembly for 17-VLT002652, and assemblies from short reads only for all other isolates. Read data were downsampled to 100× before assembly. Assemblies were inspected using Bandage [29] when necessary. Annotation of assemblies was performed using Prokka v1.14.5 [30] using the *S. enterica* type strain *S.* Typhimurium LT2 as a reference and default settings. Roary v3.13.0 [31] was run on the annotated assemblies, with a 90% identity cutoff and otherwise default settings, to identify the core and pan genomes. Scoary v1.6.16 [32] was run on the results to identify genes that were significantly more frequently observed in porpoise isolates compared to the reference isolate genomes. Structural information for the interpretation of results was accessed via the hybrid assembly of 17-VLT002652. 7-gene MLST and cgMLST were performed via Enterobase [22]. A minimum-spanning tree based on cgMLST data was generated with GrapeTree via Enterobase. Boxplots and gene maps were drawn in R 2.5.0 [33] with the gggenes package [34].

## Results

### Necropsy findings associated with *Salmonella* infection

**Figure 1:**
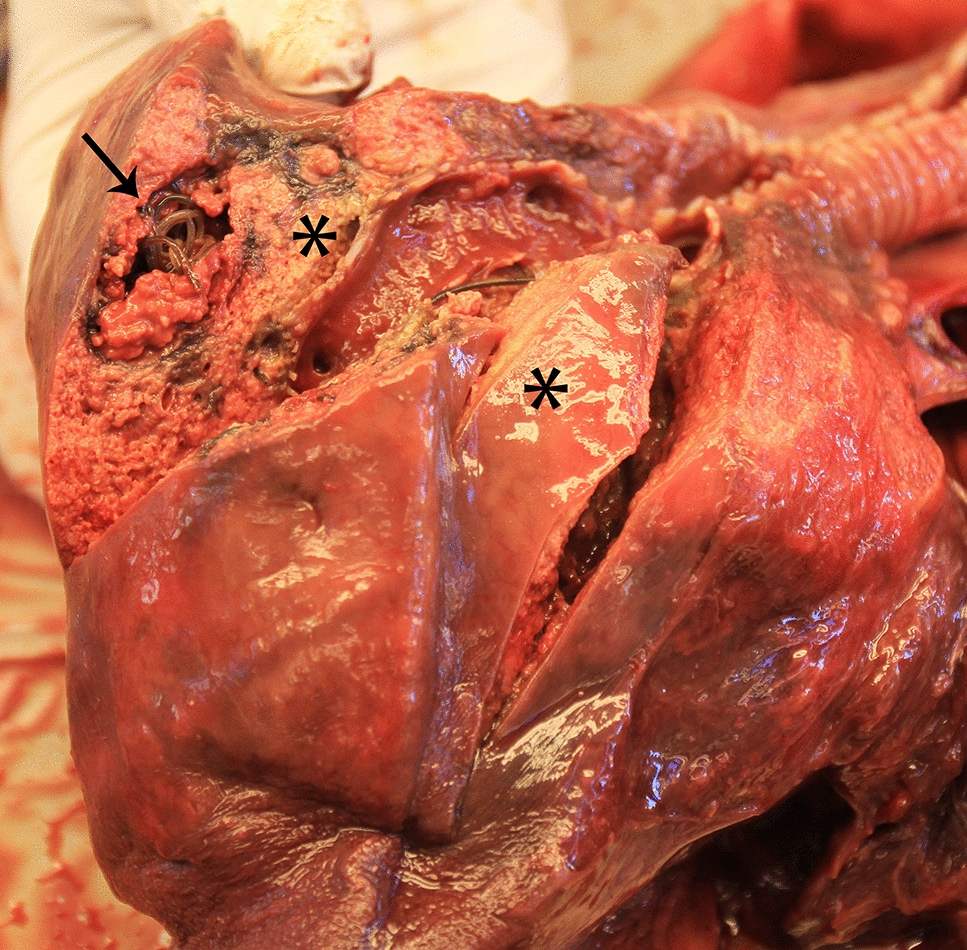
**Lung from a stranded harbour porpoise (*Phocoena phocoena*) 17-VLT002652 from which abundant growth of *Salmonella* bacteria were cultured.** Severe necrotizing pneumonia (asterisk) and moderate to heavy lungworm infestation (arrow) are evident.

**Figure 2:**
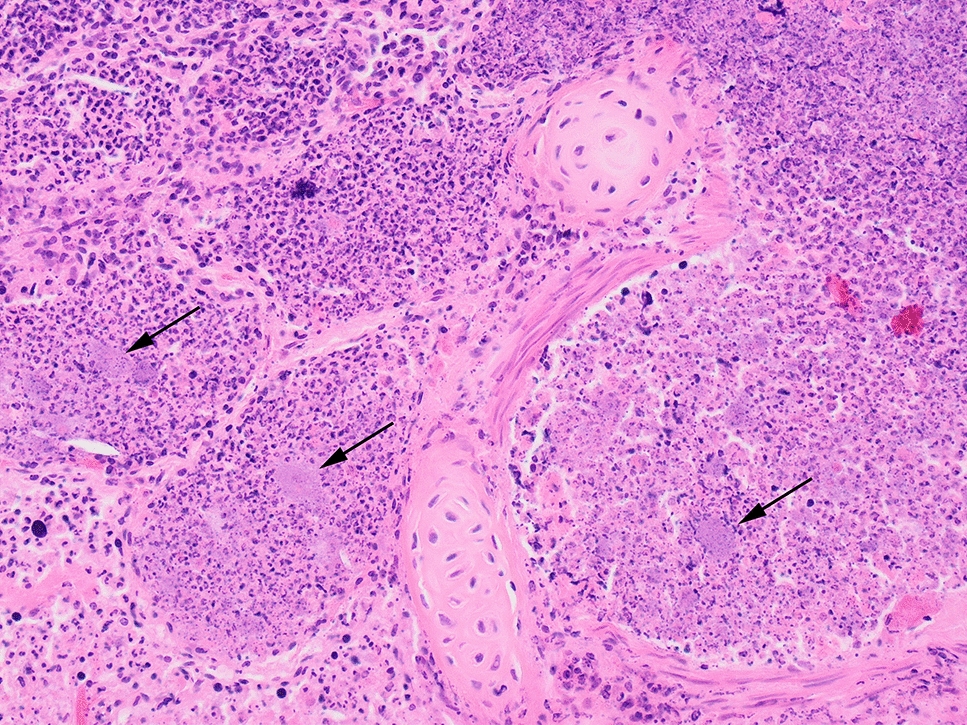
**Photomicrograph of the lung from a stranded porpoise (*Phocoena phocoena*) 17-VLT002652 displaying severe, suppurative and necrotizing bronchopneumonia.** Mats of bacteria (arrows) can be seen amongst abundant, degenerate neutrophils which fill alveoli and airways. Hematoxylin and eosin stain, ×200 magnification.

The first *Salmonella* isolate in the present study (17-VLT002652) was recovered from a porpoise found stranded in Varberg municipality (N 57°7.1′, E 12°11.5′) on the Swedish west coast in July 2017. This mature, 18-year-old male was in very poor nutritional condition and suffered from severe bronchopneumonia in which 50–60% of the lung tissue was necrotic and inflamed (Figure 1). There was a concurrent, moderate to heavy nematode infection in the airways with few parasites in the heart and pulmonary vessels. Microscopically, large areas of acute pyogranulomatous inflammation and necrosis consistent with acute bacterial pneumonia were seen and within some areas, bacteria could also be identified (Figure 2). Parasites were not associated with these lesions. Given the pathological changes seen associated with bacteria and the abundant growth of *Salmonella* from lung tissue, pneumonia caused by *Salmonella* infection was determined to be the cause of death. The recovered *Salmonella* isolate could not be serotyped and was biochemically atypical (lysine decarboxylase negative). The second isolate (20-VLT001389) was cultured from an animal found dead in February 2020 in Helsingborg (N 56°2.7′, E 12°41.2′), further south on the west coast. This immature female porpoise was in good nutritional condition and deemed to be in good health. Linear marks on the head and extremities were consistent with net marks and death was attributed to incidental capture in fishing gear. This animal had a moderate burden of nematodes in the airways and scattered areas of caseous inflammation in the lung tissue. Microscopically, focal areas of chronic eosinophilic and granulomatous pneumonia typical of parasitic infection were seen associated with nematodes. No lesions consistent with bacterial pneumonia were observed and only weak growth of *Salmonella* was observed following culture of the lung. The presence of *Salmonella* bacteria in the lungs of this animal was not associated with any pathology. This second isolate was also lysine decarboxylase negative and was serotyped as 4:a:-.

### Core-genome MLST comparison of porpoise *Salmonella* and identification of close relatives

**Figure 3:**
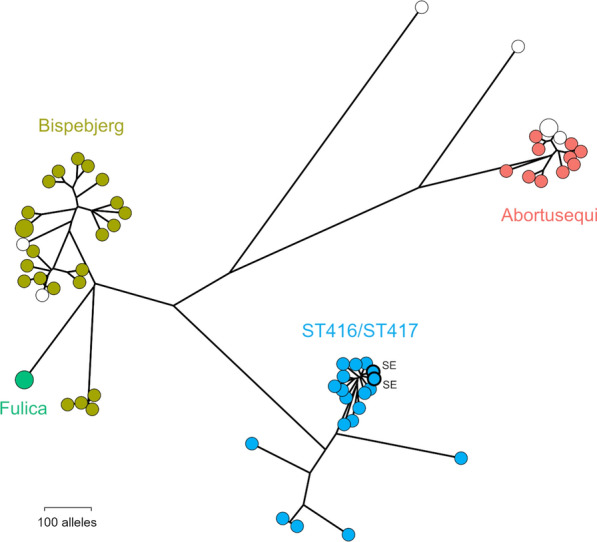
**Phylogenetic relationship between harbour porpoise isolates of *S. enterica* ST416/ST417 and their nearest neighbours as determined by cgMLST.** The closest relatives identified include isolates identified as serovars Bispebjerg, Fulica and Abortusequi. Swedish isolates sequenced in the present study are labelled SE. White circles indicate isolates for which no serotypes were reported.

Both Swedish porpoise isolates were ST416. Phylogenetic analysis based on cgMLST indicated that the Swedish isolates most closely matched 17 “Fulica-like” or “Fulica-like rough” ST416 or ST417 isolates in Enterobase, of which 16 were from harbour porpoises in Scotland and 1 was from Scotland but lacked metadata. The Swedish isolates were most similar to each other but clustered closely with the main group of Scottish isolates. Isolates of serovars Bispebjerg (*n* = 23), Fulica (*n* = 2), Abortusequi (*n* = 10) and of unknown serovars (*n* = 7) were identified as the best matches from sources other than porpoises based on belonging to the cgMLST group 4372 at the hierarchical clustering level HC2000 but were far less closely related (Figure 3). Most included Bispebjerg isolates were from humans (*n* = 12), with additional isolates from turtles (*n* = 4), food (*n* = 2), a falcon (*n* = 1) or unspecified sources. The only two isolates of Fulica, both isolated from humans, clustered together but as part of the Bispebjerg cgMLST cluster. The Abortusequi isolates were from horses (*n* = 2) or unspecified sources. The isolates without specified serotypes were from humans (*n* = 2), palm nuts (*n* = 2), a donkey (*n* = 1) or unspecified sources.

### Alterations in major virulence associated genomic regions

**Figure 4:**
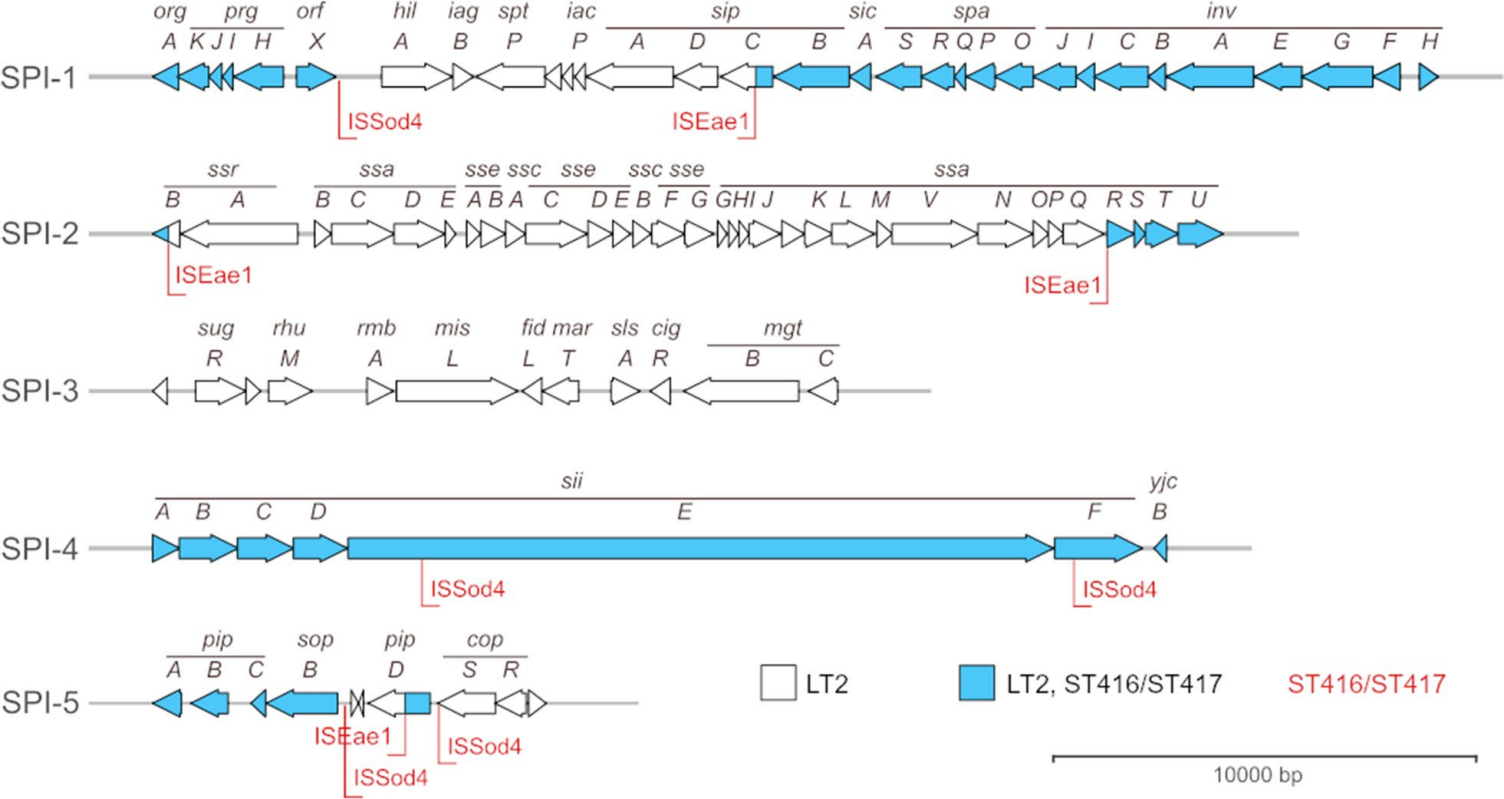
**Comparison between *Salmonella* pathogenicity islands in *S.* Typhimurium and *S. enterica* ST416/ST417.** SPI1–SPI5 (arrows) structure in the *S.* Typhimurium strain LT2 with highlighted regions present in the ST416 isolate 17-VLT2652 (blue) and IS-element insertions in 17-VLT2652 (red).

The *spvR*,*A*,*B*,*C*,*D* genes forming the signature locus of the *Salmonella* virulence plasmids [35] were absent in all ST416/417 isolates. The *spv* locus was present in most *S. *Abortusequi isolates, and a single isolate with no serovar assigned, while absent from the remaining genomes analysed. Major structural differences were observed between ST416/417 isolate pathogenicity islands SPI1-5 and those of the related isolates (Figure 4, Additional file 1). SPI-1 was found to be partially deleted with *hilA* which is a transcriptional regulator necessary for SPI-1 expression [36] missing together with *iagB* and the translocated effector proteins *sptP*, *sipA* and *sipD*. The majority of SPI-2 was also absent, leaving only *ssrB* and a segment containing *ssaU*,*T*,*S*,*R*. SPI-3 was completely absent together with a ~15kbp downstream genomic region present in LT2. SPI-4, which encodes a T1SS and a single large secreted adhesin, was present in the 17-VLT002652 genome, but both the adhesin gene (*siiE*, annotated as a hypothetical protein with locus tag STM4261 in the LT2 genome) and an essential gene for the T1SS (*siiF*, locus tag STM4262) were disrupted by IS-element insertions in coding regions. SPI-5 was also partially absent, with only *sopB* and *pipA*,*B*,*C* remaining. A short fragment consistent with a partial SPI-5 *pipD* gene was present elsewhere in the genome flanked by insertion sequence (IS)-element transposase genes. IS*256* family ISEae1 or ISSod4 element transposase genes were present flanking the described deletions. The deletions observed in SPI-1, SPI-2, SPI-3 and SPI-5 were shared among ST416/417 isolates with variation in the extent of the deletions in a few isolates (Additional file 1). In contrast, the elements were mostly conserved in Bispebjerg, Fulica and Abortusequi isolates with only minor variation in SPI-3 and SPI-5 (Additional file 1). Consistent with the changes in SPIs described above, KEGG pathway analysis indicated overrepresentation of alterations in the categories “*Salmonella* infection”, “Bacterial secretion system” and “Bacterial invasion of epithelial cells” (Table 1) in the ST416 isolate 17-VLT002652 compared to the *S. enterica* type strain LT2.

**Table 1:** **KEGG pathways altered in ST416/ST417**

Pathway	Pathway name	Pan-genome	SNP
stm01100	Metabolic pathways	46	30
stm01120	Microbial metabolism in diverse environments	35	9
stm02020	Two-component system	20	9
stm05132	Salmonella infection	13	5
stm02060	Phosphotransferase system (PTS)	12	–
stm01110	Biosynthesis of secondary metabolites	12	7
stm03070	Bacterial secretion system	12	–
stm00350	Tyrosine metabolism	10	2
stm00030	Pentose phosphate pathway	9	–
stm01220	Degradation of aromatic compounds	7	–
stm01200	Carbon metabolism	6	2
stm02024	Quorum sensing	6	–
stm02010	ABC transporters	5	5
stm00051	Fructose and mannose metabolism	5	4
stm00520	Amino sugar and nucleotide sugar metabolism	5	4
stm00910	Nitrogen metabolism	5	–
stm00010	Glycolysis/gluconeogenesis	5	–
stm01230	Biosynthesis of amino acids	4	–
stm00310	Lysine degradation	4	3
stm05100	Bacterial invasion of epithelial cells	3	–
stm00330	Arginine and proline metabolism	3	–
stm00630	Glyoxylate and dicarboxylate metabolism	3	2
stm00650	Butanoate metabolism	3	2
stm00680	Methane metabolism	3	2
stm00620	Pyruvate metabolism	3	–
stm00260	Glycine, serine and threonine metabolism	3	–
stm03430	Mismatch repair	3	–
stm00760	Nicotinate and nicotinamide metabolism	–	3
stm00250	Alanine, aspartate and glutamate metabolism	–	3
stm01503	Cationic antimicrobial peptide (CAMP) resistance	-	3
stm03010	Ribosome	2	–
stm00071	Fatty acid degradation	2	–
stm00400	Phenylalanine, tyrosine and tryptophan biosynthesis	2	–
stm00633	Nitrotoluene degradation	2	–
stm00640	Propanoate metabolism	2	–
stm00500	Starch and sucrose metabolism	2	4
stm02030	Bacterial chemotaxis	2	-
stm00564	Glycerophospholipid metabolism	2	–
stm00562	Inositol phosphate metabolism	2	–
stm00541	O-Antigen nucleotide sugar biosynthesis	–	2
stm00540	Lipopolysaccharide biosynthesis	–	2
stm02060	Phosphotransferase system (PTS)	–	2
stm00052	Galactose metabolism	–	2
stm00230	Purine metabolism	–	2

KEGG pathways significantly enriched for presence/absence variation in isolates of ST416/ST417 compared to the *S. enterica* type strain LT2 as determined by pan-genome analysis and identification of high impact (i.e., presumed loss of function) SNPs.

### Alterations in genes encoding metabolic pathways and other biological functions

ST416/417 isolates were shown by pangenome and SNP analysis to have enriched loss-of-function alterations in several metabolic pathways when compared to LT2 (Table 1). Genes associated with microbial metabolism in diverse environments were particularly strongly affected, as were pathways involved in sensing and responding to environmental cues such as two-component systems and quorum sensing. Contrasting ST416/417 with its close relatives by pangenome analysis revealed that several of the ST416/417 vs. LT2 differences were not shared with the rest of group 4372 (Additional file 2). For example, ST416/417 appear to consistently lack the gene encoding glucarate dehydratase (*gudD*) which is part of a locus essential for fermentation of galactarate and glucarate in *S*. Typhimurium. Loss of function in this locus is a trait known to be common only among serovars associated with extraintestinal infection [37]. Several genes of the 4-hydroxyphenylacetate catabolic pathway were missing in ST416/417, consistent with pseudogene formation in this locus in *S*. Typhi [17], as were the formate hydrogenlyase genes *hycA*,*B*,*C*,*D* which are active under anaerobic conditions [38]. Also missing was the transcription factor *eutR*, which is involved in niche recognition and utilization of ethanolamine in the inflamed host intestine by acting as a sensor for this substance [39]. Loss of *eutR* has previously been linked to host adapted extraintestinal pathovars of *Salmonella* [18]. Furthermore, multiple genes involved in adhesion, chemotaxis and motility (*stdA*,*B*,*C*; *fljA*; *tsr*, *hin*) which have been described as frequently disrupted in extraintestinal serovars [18] were absent or significantly altered in ST416/417, as was interestingly the aerotaxis receptor gene *aer*.

### Acquired accessory genes

ST416/417 were found to be in possession of a number of genomic regions absent elsewhere in group 4372 (Additional file 2). Several plasmid and prophage-associated genes were included in this unique set, and PlasmidFinder-2 identified three contigs as putative plasmids of incompatibility types IncI1-I(Gamma) (contig 26), IncFIB (contig 3) and IncFII (contig 19). Bandage analysis confirmed that contigs 26 and 19 were indeed consistent with circular plasmids, whereas contig 3 appears to be chromosomal. Notably, the *iucA*,*C*,*D*/*iutA* aerobactin gene cluster encoding a siderophore iron uptake system associated with extraintestinal infections by both avian pathogenic *E. coli* (APEC) [40] and poultry-associated *Salmonella* [41] was uniquely present in all ST416/417. The same was true for the *tsh* gene encoding a temperature-sensitive hemagglutinin which is also linked to APEC virulence [42], and which was co-located with *iucA*,*C*,*D*/*iutA* in 17-VLT002652. ST416/417 also uniquely carried a version of the *hemR* hemin receptor gene, encoding another putative iron uptake system.

### Model-based estimation of extraintestinal niche adaptation

**Figure 5:**
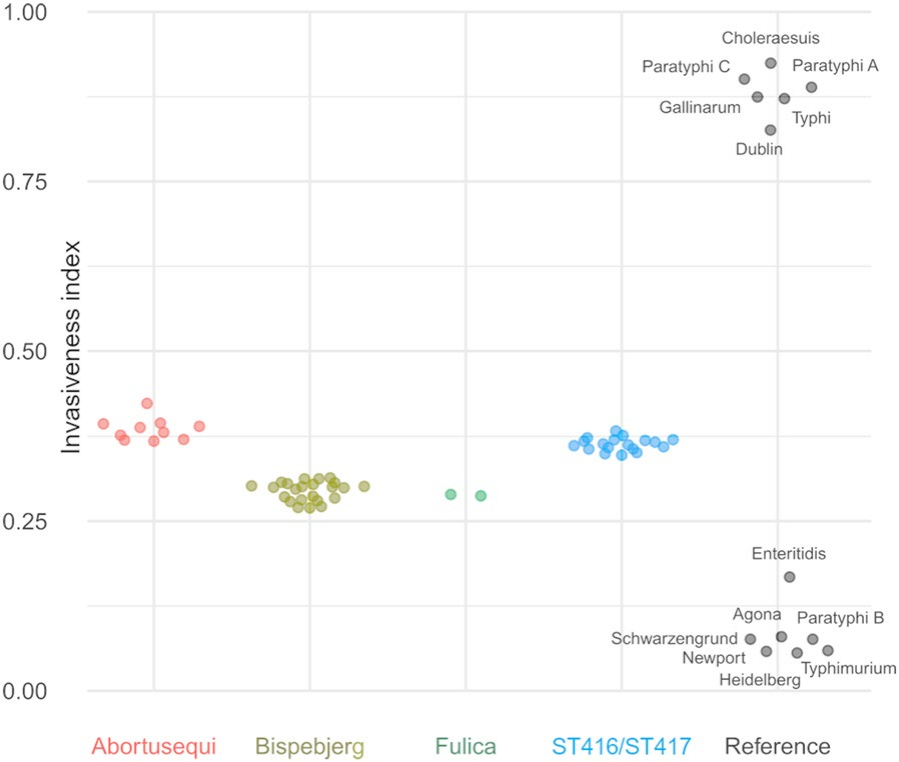
**Invasiveness index for *Salmonella* isolates analysed in the present study compared to reference serotypes.** Reference isolates considered to be associated with gastrointestinal (low score) or extraintestinal (high score) infection shown to the right.

A random forest classifier model previously trained to identify strains associated with extraintestinal disease based on well-characterised gastrointestinal and extraintestinal *Salmonella* serovars [27] was used to assess all ST416/417 isolates and their closest relatives. The results indicate that all group 4372 have genetic adaptations previously observed in extraintestinal serotypes, with ST416/417 and Abortusequi scoring higher than Bispebjerg and Fulica (Figure 5). However, all tested isolates scored lower than the reference set of extraintestinal serovars.

## Discussion

We here report the first observations of putatively host-adapted group B *Salmonella enterica* ST416 in harbour porpoises from Swedish waters. Both infected animals were found in a region occupied by porpoises of the Belt Sea population, but genetic analysis would be necessary to confirm their origin. Unlike studies on porpoises from waters around Great Britain where confirmed or presumed ST416/417 have been isolated from 20 to 35% of porpoises analysed [11, 12, 43], this bacterium was found in only 2 of the 47 lung samples (4%) examined in this study. However, relatively few porpoises were examined, and isolation rates may have been influenced by isolation protocols, freezing of carcasses prior to examination, and the effects of post-mortem decomposition. The occurrence of closely related strains in Swedish and British porpoises supports the theory of ST416/417 as specifically adapted for cetacean hosts. To our knowledge and with the exception of one possible case described from the Netherlands [6], host-adapted *Salmonella* has not been reported in harbour porpoises elsewhere. In a previous study, a few *Salmonella* sp. isolates were recovered from porpoises in the German Baltic Sea, but no infection was observed in animals from the German North Sea or in the waters around Norway and Greenland [44]. The recovered *Salmonella* isolates were not typed. Interestingly, Valderrama Vasquez et al. [43] found a significant increase in the frequency of *Salmonella* infection among porpoises over time from 6% in 1990–1994 to 27% in 2000–2002. The reason for this increase is not known but change in immune competence of the host, for example due to anthropogenic environmental disturbances, cannot be excluded. Further surveillance is needed to better estimate the prevalence of ST416/417 among Swedish porpoises and to monitor changes over time, as well as to investigate possible links between parasite load and risk of salmonellosis for these animals. Recovering more isolates for whole-genome analysis would also be beneficial to determine if ST416/417 has been introduced recently in Swedish waters, as we would expect a long-term established wildlife reservoir to host a higher degree of genetic variation among the bacteria.

Our results show that ST416/417 is not necessarily pathogenic for porpoises, as it was cultured from the lungs of an apparently healthy animal in the absence of associated pathology. However, it did cause serious disease in the other case. This is consistent with findings in other studies [10, 11, 43]. These researchers propose that this type of *Salmonella* is part of the commensal flora in the lung of porpoises but may become an opportunistic pathogen under the right conditions, including immunosuppression. The major genetic virulence factors of *S. enterica* are encoded on pathogenicity islands, SPIs, and we here show that the key SPIs 1–5 appear to be largely inactivated in ST416/417 in contrast to those of near relatives. These SPIs are believed to have been acquired early in the evolution of *S. enterica* and most of them are generally considered to be highly conserved among well studied serovars [15, 45]. It is possible that extensive loss of virulence factors is a common adaptation among commensal and opportunistically pathogenic strains of *S. enterica*, which are less likely to be sampled and sequenced. We note that the inactivation of these elements seems to have been the result of IS element activity, a mechanism of virulence modulation known to occur in a wide range of bacterial species [46].

Gastrointestinal serovars of *S. enterica* have extensive metabolic capabilities evolved e.g. to exploit nutrients available in the inflamed host gut. The complex genetic network needed for this tends to be degraded in previously studied extraintestinal serovars [17, 18]. Pathway analysis indicates reduced metabolic function in ST416/417, consistent with the fact that this type of *Salmonella* has in most cases been recovered from extraintestinal samples and in particular from the lungs of infected porpoises. ST416/417 also share other traits common among extraintestinal serovars such as loss of capabilities for sensing and responding to environmental cues. Directly linking alterations contributing to virulence or host adaptation on a gene-by-gene basis is complicated by our limited understanding of the complex networks of gene interaction that determine the fitness of a strain under a particular set of circumstances. Approaches based on machine learning show great promise in solving this type of problem and have recently been applied to *Salmonella* classification and source attribution [27, 47, 48]. The system implemented in the present study classified ST416/417 isolates as moderately invasive, but the small size and diversity of the training dataset is likely to be a limiting factor. Future improvement of predictions of host bias or disease phenotype based on genomics will strongly depend on the continued collection of high-quality epidemiological and clinical outcome data as well as sequences.

Unsuccessful attempts have been made in the past to find close relatives of the porpoise *Salmonella* strains using MLST [9], and other molecular methods such as ribotyping, insertion sequence element fingerprinting and PCR-based profiling [49]. Our results using cgMLST largely reflect the results of these previous studies in not being able to find any closely related isolates from other sources, despite the substantial number of genome sequences now available for cgMLST comparison in Enterobase. Again in agreement with results from the aforementioned studies, the closest relatives we do find include some but not all serotypes with similar antigenic profiles to 4,12:a:- (Figure 3). Bispebjerg (1,4,[5],12:a:e,n,x) and the rare Fulica (4,[5],12:a[1,5]:) are both poorly characterized and seem to occur in diverse sources including cases of illness in humans. The somewhat less closely related Abortusequi (4,12:-:e,n,x) is host restricted to horses [13] and known for causing extraintestinal infections in the form of equine paratyphoid characterized by abortions in mares and septicaemia in young foals [50]. Interestingly, Abortusequi has been recovered in high numbers from parasitic aneurysms in apparently healthy and otherwise *Salmonella*-negative horses [51], with the hypothesis raised that the lesions provided a niche for long-term carriage of the bacteria. An outlier without serotype assignment among the identified related strains was recovered by metagenomic sequencing from the tooth pulp of a Neolithic hunter-gatherer, presumably suffering from systemic infection [52]. In general, a parallel can perhaps be drawn between this lineage of *S. enterica* and that containing the generalist serovar Enteritidis, the host-adapted Dublin (cattle) and the host-restricted Gallinarum (poultry) [15] with a set of shared adaptations providing evolutionary opportunities for further specialization for different hosts. We intend to further investigate this by targeted sequencing of historical *Salmonella* isolates with antigen profiles similar to 4,12:a:- from other wildlife sources.

As previously mentioned, further studies will also be necessary to determine the relevance of extraintestinal salmonellosis as a health threat for porpoises and the possible emergent health effect of the combination of microbial infections, parasite load and environmental stressors. While the public health relevance of porpoise *Salmonella* strains is likely to be low due to limited exposure and likely reduced virulence, other host adapted serovars of *Salmonella* including Dublin [53], Choleraesuis [54], and certain Typhimurium [55] commonly cause illness in humans. Adaptation for causing extraintestinal infection in some of these serovars in fact increases the risk of severe and life-threatening systemic *Salmonella* infections, generally affecting the elderly or otherwise vulnerable [53, 54]. A better understanding of the genetic changes underlying the evolution of host adaptation and extraintestinal niche specialization therefore has the potential to be highly valuable both for improving animal and human health.

## Supplementary Information

**Additional file 1.** **Deletions in Salmonella pathogenicity islands in** ***S. enterica*** **ST416/ST417.** Presence (black) or absence (grey) of genes in *Salmonella* pathogenicity islands SPI-1,2,3,5 as determined by pangenome (Roary) analysis. The two Swedish isolates from the present study are labelled SE. Extensive deletions are evident in all ST416/ST417 isolates, while the islands are highly conserved among other related strains.

**Additional file 2.** **Pan-genome analysis output, presence/absence and significance testing.** Number of isolates of ST416/ST417 (referred to as “positive”) and other related *Salmonella* isolates (referred to as “negative”) that were found by pan-genome analysis to posess each identified sequence, i.e. gene or gene variant, and significance testing results for comparison of the frequence of occurrence in the two groups.

**Additional file 3.** **Pan-genome analysis output, FASTA reference sequences.** FASTA format sequences for the “groups” of sequence variants compared in Additional file 2.

**Additional file 4.** **R script for processing SNP data.** A short script for the R software environment used to process and filter SNP data.

## Data Availability

Sequence data from long and short read sequencing with metadata have been deposited in the European Nucleotide Archive [56] under project accession number PRJEB40444. Typing data from cgMLST analysis has been deposited in Enterobase under isolate IDs (uberstrains) SAL_EB4912AA and SAL_EB4913AA. Pan-genome analysis output is presented in Additional files 2 and 3.

## Abbreviations

bp: base pairs
BG agar: brilliant green agar
cgMLST: core genome multi-locus sequence typing
IS: insertion sequence
MALDI-TOFMS: matrix assisted laser desorption ionization-time of flight mass spectrometry
MLST: multi-locus sequence typing
MSRV agar: modified semi-solid Rappaport–Vassiliadis agar
PCR: polymerase chain reaction
SNP: single nucleotide polymorphism
SPI: *Salmonella* Pathogenicity island
ST: sequence type
SVA: Swedish National Veterinary Institute
T1SS/T3SS: type 1/type 3 secretion system
XLD agar: xylose lysine deoxycholate agar
