# Supplementary figures and images for: Genomic signatures of host adaptation in group B Salmonella enterica ST416/ST417 from harbour porpoises
Source: Vet Res. 2021 Oct 21;52:134. doi: 10.1186/s13567-021-01001-0 (PMC8529817; doi:10.1186/s13567-021-01001-0)

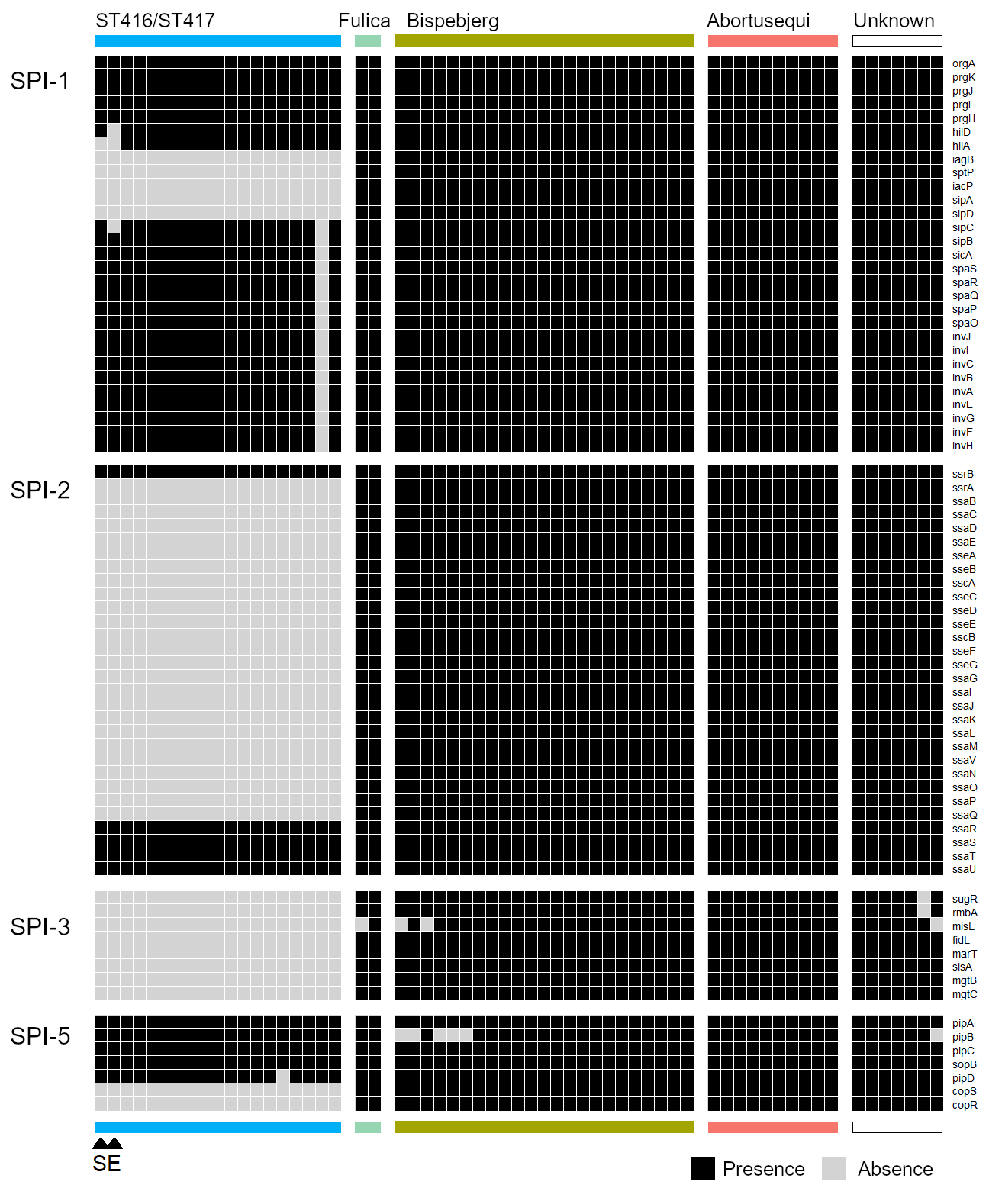

Supplement: Supplementary file 1 — Additional file 1. Deletions in Salmonella pathogenicity islands in S. enterica ST416/ST417. Presence (black) or absence (grey) of genes in Salmonella pathogenicity islands SPI-1,2,3,5 as determined by pangenome (Roary) analysis. The two Swedish isolates from the present study are labelled SE. Extensive deletions are evident in all ST416/ST417 isolates, while the islands are highly conserved among other related strains. [file 13567_2021_1001_MOESM1_ESM.png]
